# RPMB predicts the disease-free survival of head-and-neck squamous carcinoma after adjuvant concurrent radio-chemotherapy

**DOI:** 10.3389/fgene.2025.1585970

**Published:** 2025-06-11

**Authors:** Ning An, Yongchun Zhang, Haijun Lu, Xue Yang

**Affiliations:** ^1^ Department of Radiation Oncology, The Affiliated Hospital of Qingdao University, Qingdao, Shandong, China; ^2^ Department of Medical Oncology, the Affiliated Hospital of Qingdao University, Qingdao, Shandong, China

**Keywords:** head and neck squamous carcinoma, DNA repair genes, promotor methylation, adjuvant radiotherapy, disease-free survival

## Abstract

**Purpose:**

Currently, adjuvant concurrent radio-chemotherapy (ACRT) is the standard of care for head-and-neck squamous cell carcinoma (HNSC) with microscopically involved surgical margins (MISM) and/or extra-nodal extension (ENE), whereas the toxicity of cisplatin-based chemotherapy is non-negligible.

**Methods:**

In the current study, we identified a novel biomarker, referred to as RPMB (Repair Gene Promoter Methylation Burden), with the aim of identifying the subset of HNSC patients who are likely to respond favorably to ACRT. RPMB is defined as the proportion of methylated DNA repair genes (DRGs) relative to the total number of DRGs. We obtained the methylation profiles and clinical data for 528 HNSC patients from the Cancer Genome Atlas (TCGA) database.

**Results:**

Analysis revealed that the DRGs in HNSC were significantly hypomethylated compared to the other genes across the entire methylation profile (all *p*-values <0.001). HNSCs with higher RPMB tended to be ≥70 years old, female, and with primary anatomic location of oral cavity. High RPMB was found significantly related to a poor DFS in HNSCs in subgroup analysis (HR = 1.475, *p* = 0.024, 95% CI: 1.053–2.065). Moreover, Kaplan-Meier analysis showed that high RPMB was significantly associated with a poor DFS in these patients who received ACRT due to MISM or ENE (HR = 2.721, 95% Cl: 1.094–6.767, *p* = 0.025). The median DFS for patients with lower RPMB was 2.33 years (95% CI: 1.07 to +∞ years) and median DFS for those with higher RPMB was 0.64 years (95% CI: 0.62 to +∞ years).

**Conclusion:**

Low RPMB might serve as a promising indicator for identifying suitable HNSC patients who might be medically fit for ACRT.

## 1 Introduction

Head-and-neck cancer (HNC) is accountable for a great cancer burden. It is estimated about 66,920 new cases of HNCs, such as laryngeal, pharyngeal, and oral cavity cancers, will occur in 2023, accounting for up to 3.4% of new cancer cases and 15,400 deaths in the United States (Siegel et al., 2023). Head-and-neck squamous cell carcinomas (HNSCs) approximately constitutes more than 90% of HNCs. Before the milestone randomized clinical trials (RCTs) of the US Intergroup trial (RTOG 9501) and the European trial (EORTC 22931), in the majority of clinical centers, adjuvant radiotherapy (ART) was traditionally performed following the primary resection of locally advanced HNSCs. Despite the use of a relatively aggressive bimodality treatment approach, the outcomes for this treatment regimen resulted in locoregional recurrence, distant metastasis, and a 5-year overall survival (OS) rate of 30%, 25%, and 40%, respectively, implying that more aggressive treating modalities might be needed for HNSCs with certain adverse risk factors (Hong, 1992).

Therefore, these two aforementioned RCTs attempted to specify the role of adjuvant concurrent radio-chemotherapy (ACRT) in HNSC patients, including cancers of larynx, oropharynx, oral cavity, and hypopharynx, with adverse prognostic risk factors (Cooper et al., 2004; Bernier et al., 2004). Both studies compared ACRT (cisplatin 100 mg/m^2^ on days 1, 22, and 43) and ART alone in HNSCs with high-risk factors. Radiotherapy in both arms consisted of 60 Gy with or without a 6-Gy boost (RTOG) or 66 Gy (EORTC) delivered through a conventional fractionation of five fractions a week. However, the major difference between these two trials was the definition of “high-risk.” In the EORTC trial, the high risk was defined as the presence of microscopically involved surgical margins (MISM, 5 mm or less adjacent to normal tissues), extra-nodal extension (ENE) of nodal disease, the involvement of lymph nodes at levels IV or V in cancers of the oral cavity or oropharynx, perineural disease, and vascular embolism. While the RTOG trial defined high risk as positive surgical margins, ENE, and involvement of two or more lymph nodes.

The extended follow-up of the RTOG 9501 trial revealed that there were no notable differences in outcomes when comparing all randomized eligible patients. However, when analyzing a specific subgroup of patients who had either positive surgical margins or extranodal extensions (ENEs), there was an improvement in local-regional control (LRC) and disease-free survival (DFS) with the addition of ACRT (Cooper et al., 2012). Whereas a significant improvement in survival and the other prognostic parameters was observed in the EORTC trial. Of note, a combined analysis of these two trials suggested that HNSCs with MISM and ENE of cervical lymph nodes were medically fit to receive ACRT after radical surgery, while patients with other risk factors mentioned in these two trials, including stage III–IV disease, perineural invasion, lymphovascular invasion, and the involvement of level IV–V lymph nodes arising from tumors of the oral cavity or oropharynx seems to be less important (Bernier et al., 2005). Thus, most patients with MISM and ENE were recommended to receive ACRT after surgery in accordance with current clinical evidences (Peters et al., 1993; Mirimanoff et al., 1985; Feldman and Fletcher, 1982).

Due to the paucity of current evidence, there is a critical requirement for a molecular biomarker that can discern those patients who might benefit more likely from ACRT, and thereby avoiding the toxicity associated with this treatment for those who might not. Unfortunately, no such a biomarker has been discovered to fulfil the mission.

The Cancer Genome Atlas (TCGA) database is a renowned source for providing open-access datasets that encompass a wide range of cancer types (Tomczak et al., 2015). Although TCGA has been criticized as to the scarcity of its clinical data, we can still fortunately collect HNSC patients with high-risk clinical features undergoing ART or ACRT after radical surgery. The objective of this current study was intended to establish a novel predictive biomarker, referred to as RPMB (DNA Repair Gene Promoter Methylation Burden), by assessing the methylation status of DNA repair genes (DRGs), in order to find the potential subgroup of HNSCs medically fit for ACRT. Our previous investigations have shown that RPMB is notably linked to the prognoses of several cancer types, including papillary thyroid cancer (An and Yang, 2022), gastric adenocarcinoma (An et al., 2020), and non-small cell lung carcinoma (An and Yang, 2023). These findings suggested that RPMB might be an effective biomarker that could be prognostically relevant across different cancer types and treatment approaches.

## 2 Materials and methods

### 2.1 Data acquisition

The methylation profiles and associated clinical data for HNSC patients were downloaded from the Bioconductor “RTCGA” package. This dataset included methylation data from 528 primary tumor samples, which were assayed using the Illumina HumanMethylation450 chip array. Methylation value for each CpG site was pre-processed and presented as a β value, calculated as the proportion of the fluorescence intensity of the methylated allele to the total intensity of both the methylated and unmethylated alleles. This β value ranges from 0 (indicating no methylation) to 1 (indicating full methylation). A gene’s promoter region was defined as the genomic range extending from 1,000 base pairs upstream of the transcription start site (TSS) to 300 downstream. When a gene’s promoter region contained a single CpG site’s probe, the β value of this probe was used to represent this gene’s methylation level. In cases where multiple CpG sites fell within the same promoter region for a given gene, the average β value of these sites was taken as this gene’s methylation value. Therefore, a comprehensive promoter methylation profile was constructed for 19,326 genes in HNSC (An et al., 2015).

### 2.2 DRG collection and comparison of their methylation values with the other genes

DRGs were retrieved from the Gene Ontology (GO) database under the term “GO:0006281.” This yielded a total of 552 DRGs, and of these, 528 were represented in our HNSC methylation dataset. Subsequently, we compared the promoter methylation levels of these DRGs to those of the other genes (non-DRGs). Initially, we randomly selected 528 non-DRGs from the HNSC dataset, and repeated this process 1,000 times. Each set of randomly selected non-DRGs was then compared to the DRGs in terms of their methylation levels, using unpaired T-tests for each iteration. In addition, we then compared the methylation value of DRGs with those within the other 10 GO terms crucial for carcinogenesis. These terms included morphogenesis, cell death, apoptosis, proliferation, immune response, development, cell migration, angiogenesis, cell adhesion and secretion.

### 2.3 Calculation of RPMB values

The methylation levels for the 528 DRGs were categorized into binary states—either “methylated” or “unmethylated,” based on a threshold β value of 0.1. That is, a gene was considered “methylated” if its β value exceeded 0.1, and “unmethylated” if it was less than or equal to 0.1. The RPMB for an individual patient was then calculated as the proportion of methylated DRGs out of the total number of DRGs (n = 528).

### 2.4 Statistical analysis

All statistical analyses for this study were performed using R and Bioconductor software. The Bioconductor annotation package “org.Hs.eg.db” was utilized to retrieve genes associated with various GO terms. In baseline characteristics analysis, patients were categorized into two groups based on the median value of RPMB. Comparative analyses of these groups were conducted using χ^2^ tests for each clinic-pathological variable, as presented in Table 1. DFS was defined as the duration from the date of operation to the date of any disease recurrence, death due to any cause or the last follow-up. OS was defined as the time span from the date of operation to the date of death due to any cause or the last follow-up. Kaplan–Meier survival analysis and log-rank test were employed to assess survival differences between two patient groups, which were assigned based on the median value of RPMB. Additionally, we evaluated the treatment effects within different subgroups under consideration and illustrated the findings in forest plots. For the analysis of each subgroup, HNSCs were also assigned into two groups according to the median RPMB value. Subsequently, Cox regression analysis was performed to compute the hazard ratios (HRs) and 95% confidence intervals (CIs) for each subgroup.

**TABLE 1:** Patient baseline characteristics.

Characteristics	Low RPMB	High RPMB	χ^2^	*p*
Age (years)				
<70	221	190	7.531	**6 × 10** ^**−3**^
≥70	45	71		
Gender				
Male	245	141	93.689	**<2.2 × 10** ^**−16**^
Female	22	120		
Ethnicity				
Asian	4	7	5.121	0.077
White	221	231		
Black	31	17		
Location				
Hypopharynx	5	5	10.957	**0.011**
Larynx	74	43		
Oral Cavity	109	135		
Oropharynx	42	39		
Grade				
G1	29	34	4.151	0.126
G2	148	163		
G3-4	76	56		
pT				
T1-2	95	94	3.808	0.149
T3	62	79		
T4	101	83		
pN				
N0	126	123	0.040	0.980
N1	44	45		
N2-3	88	88		
Lymphovascular invasion				
No	115	117	2.922	0.087
Yes	74	50		
Perineural invasion				
No	113	85	3.700	0.054
Yes	80	92		
Stage				
I–II	59	61	1.940	0.379
III	48	59		
IV	151	136		

## 3 Results

### 3.1 Patient selection

**FIGURE 1:**
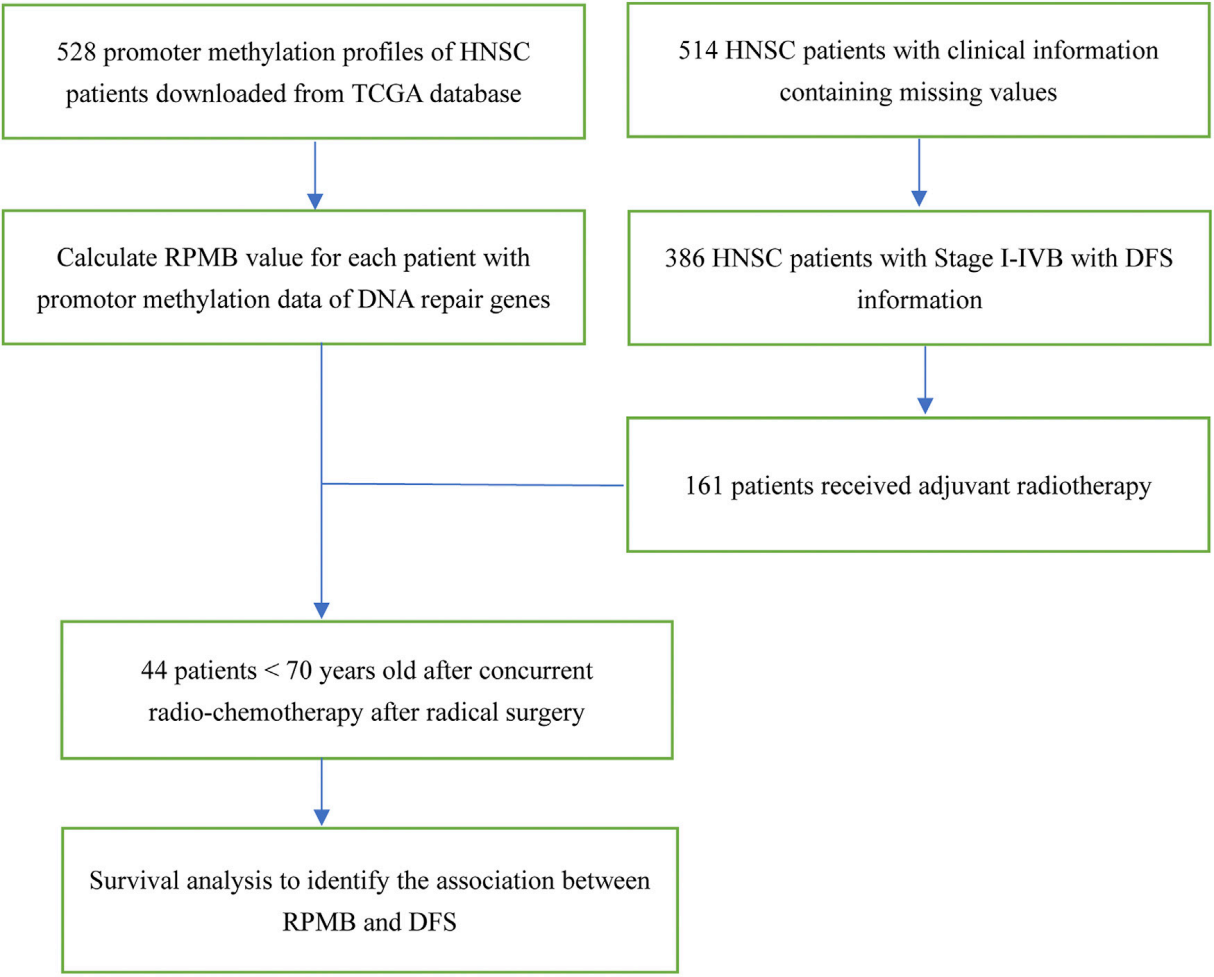
The schematic diagram of this study.

The schematic diagram is illustrated in Figure 1. TCGA HNSC clinical dataset documented the clinic-pathological information of 528 patients. We focused on the HNSC patients with local disease (Stage I–IVB, according to AJCC seventh staging system in TCGA) and confirmed having received radical surgery. Therefore, 506 patients with OS information, and 386 with DFS information were collected for further analysis. We then paid attention to HNSC patients who received ART with the following criteria: (Siegel et al., 2023): undergoing no neo-adjuvant therapy of any kind; (Hong, 1992); receiving radical surgery with adequate regional lymph node dissections; (Cooper et al., 2004); having detailed information of anatomic neoplasm subdivisions; (Bernier et al., 2004); excluding HNSCs of lips because whether cutaneous or mucosal lip was not documented. Thus, 161 HNSC patients were collected for further DFS analysis (Figure 1).

### 3.2 Methylation level of DRGs were substantially lower compared to other genes

**FIGURE 2:**
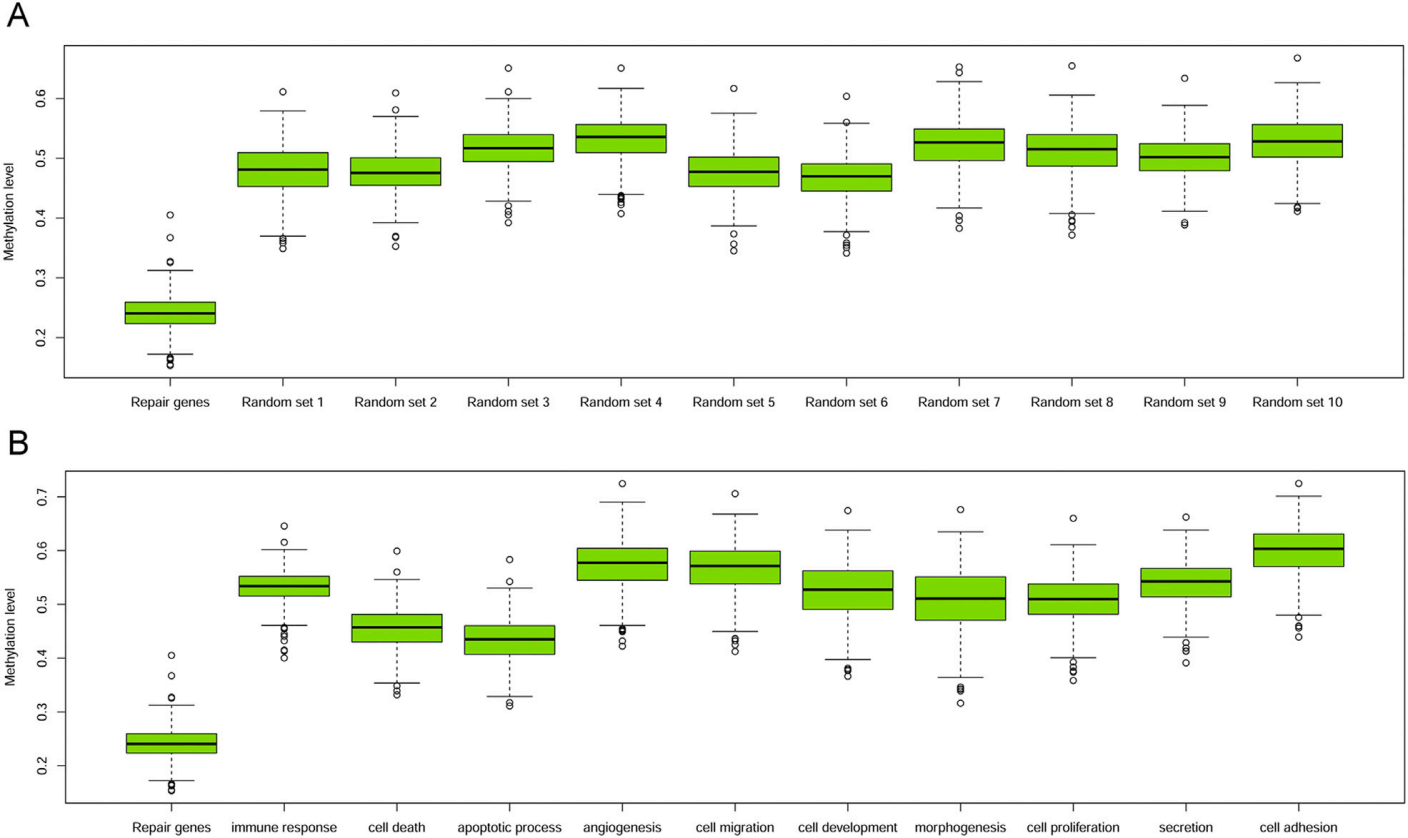
Boxplots of promoter methylation values of DRGs and others. **(A)** Boxplot of methylation levels of DRGs and other 10 sets of randomly sampled non-DRGs. **(B)** Boxplot of methylation levels of DRGs with those within other 10 GO terms.

Initially, we conducted a comparison between the methylation levels of DRGs and 528 randomly chosen non-DRGs (the remaining genes excluding DRGs), repeating this process for 1,000 times. The median methylation level for DRGs was 0.241, and unpaired T-tests indicated that DRGs’ methylation was significantly lower than that of any other randomly selected gene groups, with p-values consistently below 0.001 (Figure 2A). Furthermore, we extended our comparison to include the methylation levels of DRGs against those in 10 other biological GO terms, encompassing processes pivotal in cancer, such as morphogenesis, cell death, apoptosis, proliferation, immune response, development, cell migration, angiogenesis, cell adhesion and secretion. In each case, the promoter methylation level of DRGs was also significantly lower than that of genes involved in these biological processes (all *p* < 0.001, Figure 2B).

### 3.3 Baseline characteristics

Table 1 presents the demographic information and fundamental clinical attributes for HNSC patients. The patients were initially categorized into two groups with low and high RPMB according to the median RPMB value. A thorough analysis was performed to examine the relationship between RPMB and various clinical characteristics, which included age, gender, ethnicity, primary anatomic location, histological grade, pathological tumor size (pT), pathological regional lymph node (pN), lymphovascular invasion, perineural invasion, and pathological stage. Statistical analyses using the χ^2^ test indicated a significant association between RPMB and three key clinical factors: age, gender, and the primary anatomical site of the tumors (Table 1). Patients with high RPMB tended to be patients ≥70 years old (Wald χ^2^ = 7.531, *p* = 6 × 10^−3^), female (Wald χ^2^ = 93.689, *p* < 2.2 × 10^−16^), and with primary anatomic location of oral cavity (Wald χ^2^ = 10.957, *p* = 0.011). Additional clinical characteristics, such as ethnicity, histological grade, pT, pN, lymphovascular invasion, perineural invasion, and pathological stage, were distributed evenly between the low and high RPMB groups (*p* > 0.05).

### 3.4 Subgroup analyses in terms of OS and DFS

**FIGURE 3:**
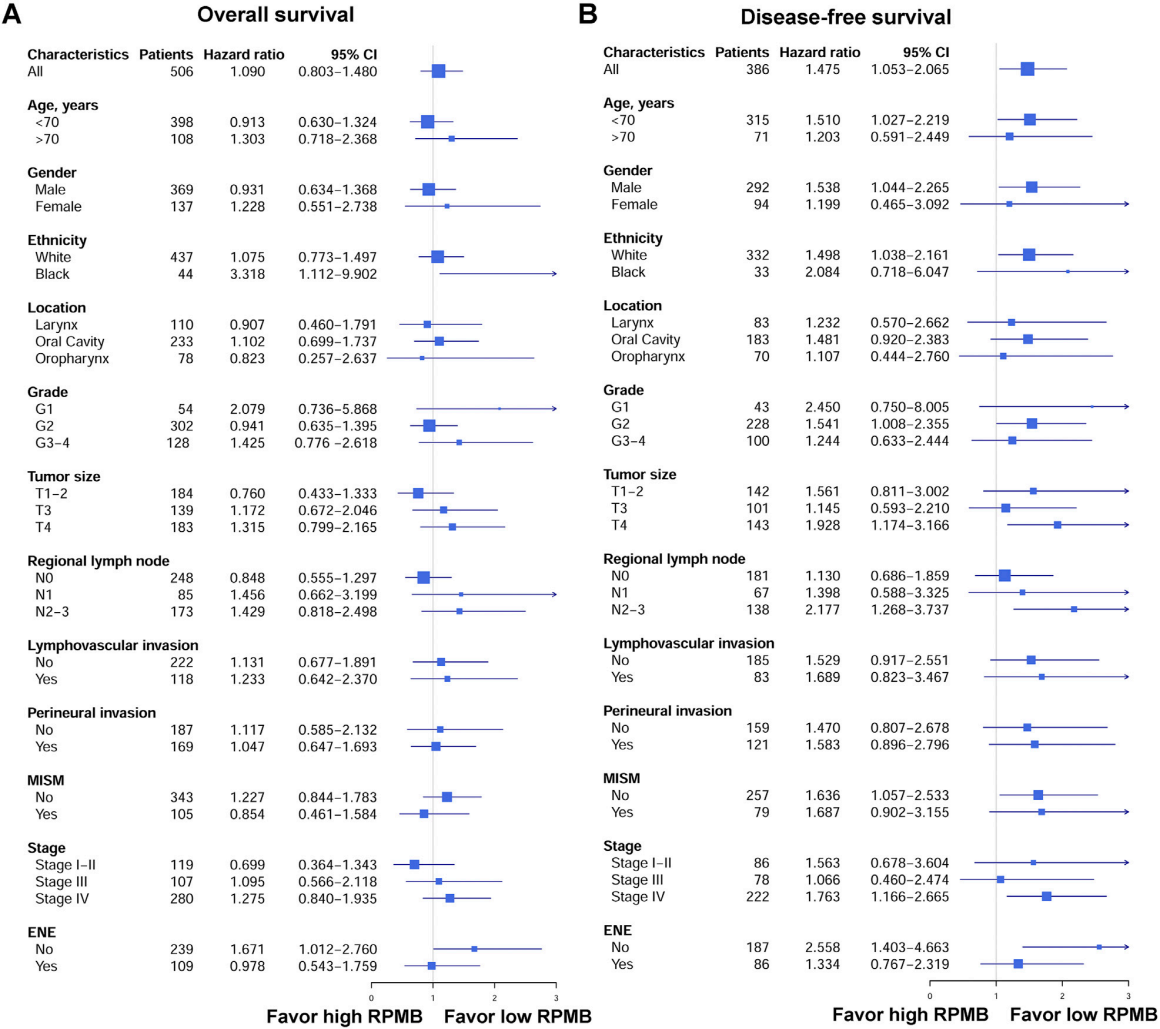
Forest plots of OS and DFS in patients subgroups. **(A)** Forest plots of OS. **(B)** Forest plots of DFS.

Figure 3 showed the forest plots of OS and DFS in subgroup analysis. First of all, we collected all the HNSC patients who received radical surgery and were pathologically staged as Stage I–IVB (local disease without distant metastasis). With regard to OS, 506 HNSCs had detailed OS information, and the HR was 1.090 for overall cohort (*p* = 0.581, 95% CI: 0.803–1.480). The subgroups were established based on clinicopathological stratification factors, including age (<70 and ≥70 years old), gender, ethnicity (white and black), anatomic location (larynx, oral cavity, and oropharynx), grade (G1, G2 and G3-4), pT (T1-2, T3 and T4), pN (N0, N1 and N2-3), lymphovascular invasion, perineural invasion, MISM, pathological stage (Stage I-Il, Stage III, and Stage IV), and ENE. The subgroups with <30 patients were excluded from this analysis. Subgroup analysis for OS indicated that none was significantly associated with RPMB, except for black people (HR = 3.318, *p* = 0.032, 95% CI: 1.112–9.902), and HNSCs with no ENE (HR = 1.671, *p* = 0.045, 95% CI: 1.012–2.760). The other subgroups did not show a clear favoring tendency. As for DFS, 386 patients were collected with detailed DFS information, and high RPMB was significantly related to a poorer DFS in HNSCs (HR = 1.475, *p* = 0.024, 95% CI: 1.053–2.065). All the subgroups favored low RPMB, and many subgroups showed a significant association with RPMB values, including patients <70 years old (HR = 1.510, *p* = 0.036, 95% CI: 1.027–2.219), male (HR = 1.538, *p* = 0.029, 95% CI: 1.044–2.265), white people (HR = 1.498, *p* = 0.030, 95% CI: 1.038–2.161), Grade 2 (HR = 1.541, *p* = 0.046, 95% CI: 1.008–2.355), T4 (HR = 1.928, *p* = 0.009, 95% CI: 1.174–3.166), N2-3 (HR = 2.177, *p* = 0.005, 95% CI: 1.268–3.737), no MISM (HR = 1.636, *p* = 0.027, 95% CI: 1.057–2.533), Stage IV (HR = 1.763, *p* = 0.007, 95% CI: 1.166–2.665), and patients with no ENE (HR = 2.558, *p* = 0.002, 95% CI: 1.403–4.663).

### 3.5 DFS and OS after ART

**FIGURE 4:**
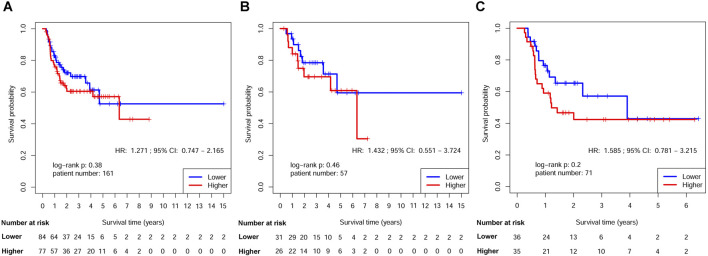
Kaplan-Meier estimates of DFS after ART by RPMB level. **(A)** Kaplan-Meier analysis in the full analysis set. **(B)** Kaplan-Meier analysis in Stage III-IVB HNSCs with no MISM or ENE. **(C)** Kaplan-Meier analysis in HNSCs with MISM and/or ENE.

**FIGURE 5:**
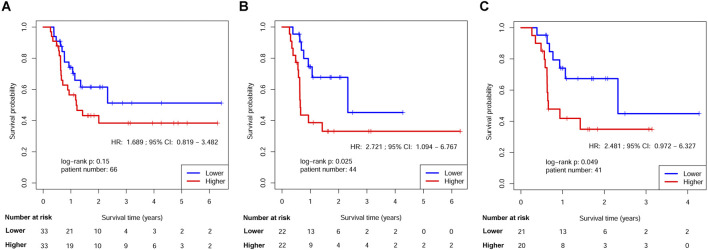
Kaplan-Meier estimates of DFS in HNSCs <70 years old after ART by RPMB level. **(A)** Kaplan-Meier analysis in the full analysis set. **(B)** Kaplan-Meier analysis in HNSCs <70 years old after ACRT. **(C)** Kaplan-Meier analysis in Stage III-IVB HNSCs <70 years old after ACRT.

The survival disparities between HNSCs with higher and lower RPMB values were assessed using Kaplan-Meier survival analysis and log-rank test. In the DFS analysis, which included 161 patients who had received ART, the HR for the association between RPMB and DFS was 1.271, with a 95% CI of 0.747–2.165. However, the observed difference in DFS between the two RPMB-assigned groups did not reach statistical significance (*p* = 0.38, as depicted in Figure 4A). Among these 161 patients, 57 HNSCs were Stage III-IVB diseases with no MISM or ENE. HR for these local advanced HNSCs was 1.432 (95% Cl: 0.551–3.724, *p* = 0.46, Figure 4B), and two survival curves were intertwined with each other. We further collected 71 patients with MISM and/or ENE for DFS analysis. Although significance was still not reached, the result revealed a clear trend toward inferior DFS among patients with elevated RPMB levels (HR = 1.585, 95% Cl: 0.781–3.215, *p* = 0.2, Figure 4C). Additionally, 66 HNSCs <70 years old (n = 66) with MISM and/or ENE showed a more prominent survival difference between high/low RPMB groups (HR = 1.689, 95% Cl: 0.819–3.482, *p* = 0.15, Figure 5A). Since the ACRT was the standard of care for HNSCs with MISM and/or ENE, we thus focused our attention upon this subgroup of patients. There were 44 HNSC patients <70 years old who received ACRT due to MISM and/or ENE. The result suggested that high RPMB was significantly related to a poor DFS in this subgroup of HNSCs (HR = 2.721, 95% Cl: 1.094–6.767, *p* = 0.025, Figure 5B). The median DFS for HNSCs with lower RPMB was 2.33 years (95% CI: 1.07 to +∞ years) and median DFS for those with higher RPMB was 0.64 years (95% CI: 0.62 to +∞ years). Among these 44 patients, 41 were Stage III-IVB HNSCs, and statistical significance was also reached for these patients (HR = 2.481, 95% Cl: 0.972–6.327, *p* = 0.049, Figure 5C).

Moreover, we also conducted OS analysis in Stage III-IVB patients after ART without MISM or ENE (Supplementary Figure 1A), patients with MISM and/or ENE (Supplementary Figure 1B), patients <70 years old with MISM and/or ENE (Supplementary Figure 1C), and HNSCs <70 years old after ACRT due to MISM and/or ENE (Supplementary Figure 1D). None of these analyses reached a statistical significance (Supplementary Figure 1).

### 3.6 Cox analyses of DFS in MISM/ENE patients after ACRT

Table 2 showed the result of univariate Cox analyses with RPMB and some other clinic-pathological factors, including age, gender, histology, pathological stage, lymphovascular invasion, and perineural invasion, in terms of DFS. Eventually, 49 HNSC patients were collected with the detailed information of aforementioned variables. The result of Cox analysis indicated that RPMB was the only prognostic factor of DFS after ACRT in MISM/ENE HNSC patients (*p* = 0.048) (Table 2).

**TABLE 2:** Cox analyses of DFS in R1/ENE patients after ACRT.

Factors	HR	(95% *CI*)	*p*
Age (years)			
<70 (n = 44)	Reference		—
≥70 (n = 5)	0.199	0.026–1.554	0.124
Gender			
Male (n = 39)	Reference		—
Female (n = 10)	0.857	0.290–2.532	0.780
Histology			
G1/2 (n = 33)	Reference		—
G3/4 (n = 13)	0.490	0.164–1.462	0.201
Stage			
I–III (n = 14)	Reference		—
IVa–IVb (n = 35)	0.967	0.389–2.403	0.942
Lymphovascular invasion			
No (n = 13)	Reference		
Yes (n = 26)	1.189	0.414–3.415	0.748
Perineural_invasion			
No (n = 17)	Reference		
Yes (n = 27)	1.785	0.685–4.656	0.236
RPMB			
Lower (n = 25)	Reference		—
Higher (n = 24)	2.408	1.007–5.756	**0.048**

## 4 Discussion

Cisplatin-based ACRT is the standard of care for HNSCs with MISM or ENE after surgery, whereas the long-term adverse effects should be paid enough attention. These toxicities of cisplatin-based chemotherapy regimens may include coronary artery vasospasm, which can be a consequence of hypomagnesemia or increased serum cholesterol levels (Chaudhary and Haldas, 2003). Furthermore, it has been observed that as many as one in four patients may experience a lasting decrease in glomerular filtration rate following treatment with cisplatin-based chemotherapy (Osanto et al., 1992). An Indian single-center study found that administering cisplatin at 100 mg/m^2^ every 3 weeks, when combined with radiotherapy for HNSC, was superior to cisplatin at 30 mg/m^2^ once a week in terms of improving LRC. However, the increased LRC must be weighed against the higher incidence of severe acute toxicities, such as hyponatremia, leukopenia, neutropenia, and lymphocytopenia (Noronha et al., 2018). Hence, the search for molecular biomarkers is crucial for the purpose of identifying HNSC patients who might be the appropriate candidates for ACRT. Unfortunately, to date, no such a biomarker has ever been discovered for this clinical purpose.

The methylation of DRGs in HNSC showed the consistent pattern with our previous studies in various cancer types (An et al., 2020; An and Yang, 2023). DRGs were significantly hypomethylated with the comparison to the rest of genes across the human genome (Figure 2). The hypothesis is quite intriguing that hypomethylation of DRGs might serve as a protective mechanism to counteract the genomic instability crisis induced by the carcinogenic process. DRG inactivation through hypermethylation might deprive the normal cells of this safeguard maneuver and drive them to a potential full-scale carcinogenesis. Therefore, if there were residue tumors which could not be removed from operation, such as MISM or ENE, these patients with high RPMB would probably have a worse clinical outcome, due to the destruction of this self-protecting bio-mechanism through DRG inactivation. Notably, another consistent finding is that female patients tended to harbor high RPMB, and the gender distinction in terms of RPMB was remarkably prominent (Table 1). As we know, the incidence rate of HNSC is much higher in men than that in women due to tobacco and alcohol consumption. However, the gender difference in prognosis of HNSCs, especially in those undergoing ACRT, has not been fully addressed in previous studies. RPMB seems a sensible linkage between this huge gender distinction and the clinical outcome of ACRT, since RPMB was the only significant DFS predictor according to Cox analysis (Table 2). Further investigations are urgently required to explore the differences in RPMB between genders in HNSC, as well as in other types of cancers. Additionally, we excluded the patient ≥70 years old during the survival analysis of ACRT. Performance status and physiologic reserve should be taken into consideration before recommending ACRT in patients with high-risk features due to the adverse toxicities. Of note, the EORTC trials also excluded patients ≥70 years old in order to eliminate the bias caused by toxicities.

The methylation of DNA promoter region has been widely reported as greatly crucial in promoting aging process (Yuan et al., 2015), the development of embryo (Law and Jacobsen, 2010), and cancer (Docherty et al., 2010; Laird, 2003; Costello and Plass, 2001; Baylin, , 1997), by demolishing the normal chromatin and DNA bio-structures (Akhavan-Niaki and Samadani, 2013). Furthermore, it has been documented that alterations in the methylation patterns of DRGs are associated with the development of cancer and the clinical outcomes across various types of cancer (Weigel et al., 2019; Teodoridis et al., 2005; Mijnes et al., 2018; Maki-Nevala et al., 2019). As we know, the most prestigious example of DRG is MGMT in glioma, which was demonstrated as a prognostic indicator to predict the therapeutic advantage of chemotherapy regimen, for example, nitrosoureas (Esteller et al., 2000) and temozolomide (Hegi et al., 2004). Besides, two RCTs both certified the prognostic advantage in glioma patients with MGMT hypermethylation after the administration of both ART and temozolomide (Stupp et al., 2005; Hegi et al., 2005). Additionally, our research team was the first to report on the predictive capabilities of RPMB in the context of ART for gastric cancer, of which higher RPMB levels were significantly linked to improved DFS following ART (An et al., 2020). However, in our present study, high RPMB was significantly related to a worse DFS in HNSC patients with MISM and/or ENE after ACRT. The seemingly contradictory predictive ability of RPMB might be explained by whether there were residual tumors after surgery. In present study, the intended patient group was the HNSC patients with MISM and/or ENE, while the intended patients in glioma and gastric cancer studies were those received radical surgery with no residue tumors. The biological theory of ART in glioma and gastric cancer after complete resection is to compromise vital macromolecules, such as the biostructure of DNA molecules. The silencing of DRGs due to hypermethylation might enhance the DNA-damaging effects of ART, potentially resulting in a better prognosis. However, in present study, on account of the residual tumor mass after incomplete resection, high RPMB would result in a rampant growth of tumor cells through genomic instability caused by DRG inactivation. The hypermethylation of DRGs might sentence a death penalty to more tumors cell by ACRT, but it could not counteract the rapid tumor growth of residual tumors due to incomplete resection. The distinct clinical settings might explain the reason why high RPMB predicted an unfavorable clinical outcome in HNSC, whereas the contrary in glioma and gastric cancer. HNSC patients with low RPMB seemed to be more suitable for ACRT if MISM and/or ENE was encountered (Figures 5B, C). Systematic therapy with combination regimens might be essential for HNSCs with high RPMB in order to rescue the patients from such a poor prognosis of median DFS only 0.64 years.

The presence of numerous missing values within the clinical dataset in TCGA and the restricted patient numbers were the major limitations in currents study. For instance, only 44 HNSCs <70 years old undergoing ACRT with MISM and/or ENE were available for DFS analysis. Thus, increasing patient number in this study is important to strengthen our conclusion. Additionally, TCGA clinical dataset has very limited information upon HPV infection status of oropharynx cancers, making the subgroup analyses of HPV+ and HPV− oropharynx cancers impossible. Furthermore, as of our knowledge, TCGA HNSC dataset is the only available dataset that includes both methylation profiles and clinical outcomes for patients undergoing ACRT. Consequently, it is currently not feasible to validate the predictive power of RPMB in another independent cohort. In the future, we plan to conduct a prospective study to further corroborate our findings and to assess the potential clinical utility of RPMB in HNSC patients.

## 5 Conclusion

Low RPMB might be considered as a promising molecular biomarker helpful in finding proper HNSC patients with MISM or ENE who medically fit for the usage of ACRT.

## Data Availability

The original contributions presented in the study are included in the article/Supplementary Material, further inquiries can be directed to the corresponding authors.
