# Supplementary material for: RPMB predicts the disease-free survival of head-and-neck squamous carcinoma after adjuvant concurrent radio-chemotherapy
Source: Front Genet. 2025 Jun 11;16:1585970. doi: 10.3389/fgene.2025.1585970 (PMC12187736; doi:10.3389/fgene.2025.1585970)
Supplement: Supplementary file 1 [file DataSheet1.docx]

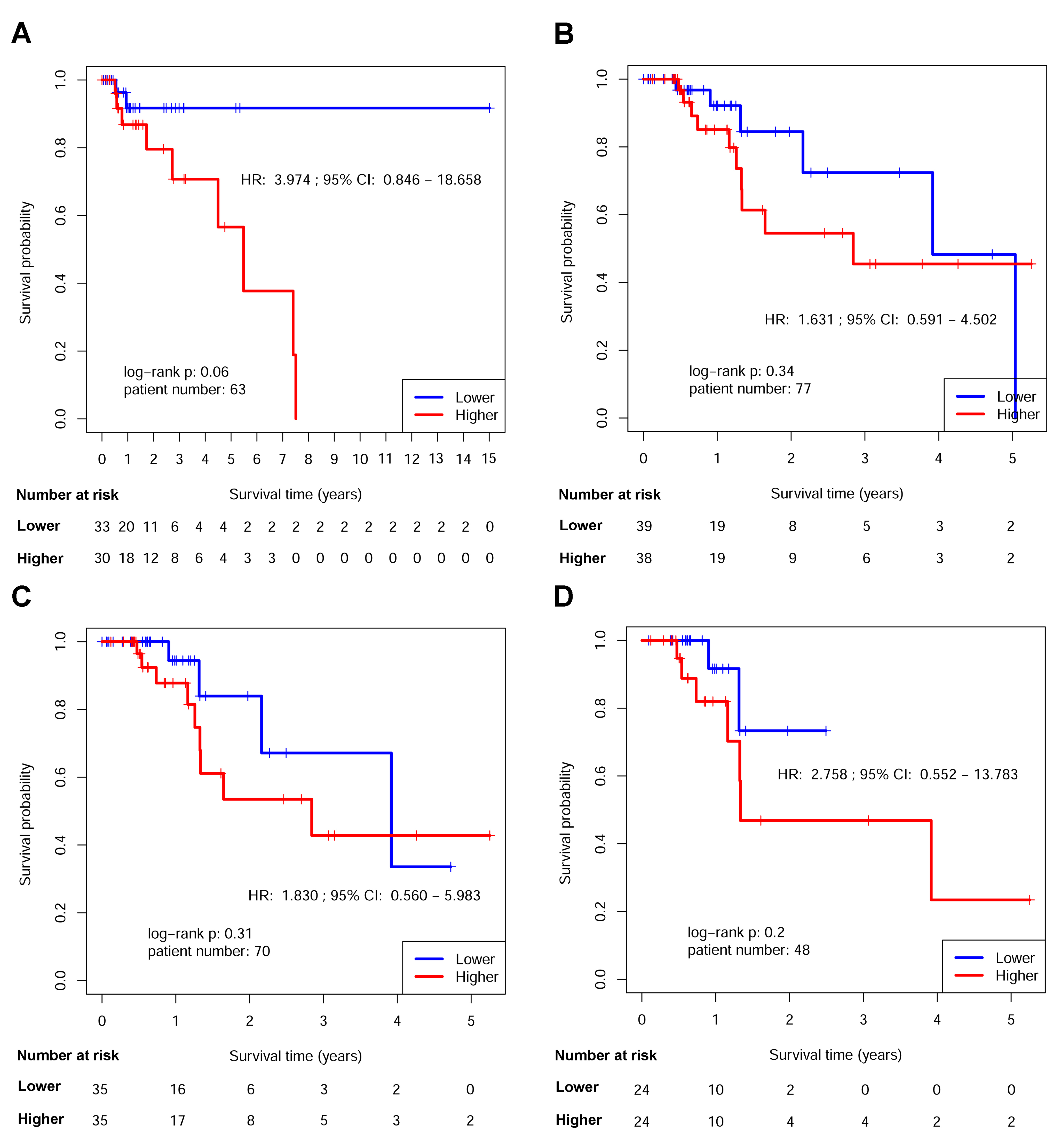


**Supplementary Figure 1.** **Kaplan-Meier estimates of OS after ART by RPMB level.** A. Kaplan-Meier analysis of Stage III-IVB HNSCs with no MISM or ENE. B. Kaplan-Meier analysis of HNSCs with MISM and/or ENE. C. Kaplan-Meier analysis of HNSCs < 70 years old with MISM and/or ENE. D. Kaplan-Meier analysis of HNSCs < 70 years old with MISM and/or ENE after ACRT.
